# Percutaneous core needle biopsy versus open biopsy in diagnostics of bone and soft tissue sarcoma: a retrospective study

**DOI:** 10.1186/2047-783X-17-29

**Published:** 2012-11-01

**Authors:** Florian Pohlig, Chlodwig Kirchhoff, Ulrich Lenze, Johannes Schauwecker, Rainer Burgkart, Hans Rechl, Ruediger von Eisenhart-Rothe

**Affiliations:** 1 Department of Orthopaedic Surgery, Klinikum rechts der Isar, Technische Universitaet Muenchen, Ismaningerstrasse 22, 81675, Munich, Germany; 2 Department of Trauma Surgery, Klinikum rechts der Isar, Technische Universitaet Muenchen, Ismaningerstrasse 22, 81675, Munich, Germany

**Keywords:** Biopsy, Open biopsy, Core needle biopsy, CNB, Sarcoma, FNA, Malignancy, Histopathology

## Abstract

**Background:**

Biopsy is a crucial step within the diagnostic cascade in patients with suspected bone or soft tissue sarcoma. Open biopsy is still considered the gold standard. However, recent literature suggests similar results for percutaneous biopsy techniques. Therefore, the aim of this retrospective analysis was to compare open and percutaneous core needle biopsy (CNB) regarding their accuracy in diagnosis of malignant musculoskeletal lesions.

**Methods:**

From January 2007 to December 2009, all patients with suspected malignant primary bone or soft tissue tumour undergoing a percutaneous CNB or open biopsy and a subsequent tumour resection at our department were identified and enrolled. Sensitivities, specificities, positive predictive values (PPV), negative predictive values (NPV) and diagnostic accuracy were calculated for both biopsy techniques and compared using Fisher’s exact test.

**Results:**

A total of 77 patients were identified and enrolled in this study. Sensitivity, specificity, PPV, NPV and diagnostic accuracy were 100% for CNB in bone tumours. Sensitivity (95.5%), NPV (91.7%) and diagnostic accuracy (93.3%) for open biopsy in bone tumours showed slightly inferior results without statistical significance (p > 0.05). In soft tissue tumours favourable results were obtained in open biopsies compared to CNB with differences regarding sensitivity (100% vs. 81.8%, p = 0.5), NPV (100% vs. 50%, p = 0.09) and diagnostic accuracy (100% vs. 84.6%, p = 0,19) without statistical significance. The overall diagnostic accuracy was 92.9% for CNB and 98.0% for open biopsy (p = 0.55). A specific diagnosis could be obtained in 84.2% and 93.9%, respectively (p = 0.34).

**Conclusion:**

In our study we found moderately inferior results for the percutaneous biopsy technique compared to open biopsy in soft tissue tumours whereas almost equal results were obtained for both biopsy techniques for bone tumours. Thus, CNB is a safe, minimal invasive and cost-effective technique for diagnosing bony lesions. In soft tissue masses, the indication for percutaneous core needle biopsy needs to be made carefully by an experienced orthopaedic oncologist with respect to the suspected entity, size of necrosis and location of the lesion to avoid incorrect or deficient results.

## Background

Sarcomas of the bone account for only about 1% of all tumour diseases [1]. Incidence rates of soft tissue sarcomas vary from 1.8 to 5.0 cases per 100,000 per year in Germany [2]. According to the UK National Institute for Health and Clinical Excellence (NICE) guidelines, patients with suspected bone or soft tissue sarcoma have to be transferred to dedicated centres for diagnostic workup and integrated therapy. Regarding diagnosis, an assessment consisting of clinical history and examination, imaging and tissue biopsy is recommended [3]. In this context, biopsy in particular is a crucial step providing the basis for any further therapeutic strategy [4]. Biopsy might be omitted only in the case of clinically and radiologically unambiguous benign lesions [5].

The different procedures such as fine needle aspiration (FNA), core needle biopsy (CNB) or open biopsy are all associated with specific advantages and disadvantages [6]. The objective of all these biopsy techniques is to gain a representative tissue sample with minimal trauma, considering the correct surgical approach for a later resection to facilitate limb-sparing procedures. Hereby, accurate preoperative planning based on the diagnostic findings is the crucial and most demanding part [5].

Open biopsy has for a long time been considered the gold standard for the diagnosis of malignant and uncertain tumours of the musculoskeletal system [4,5,7]. However, recent studies increasingly suggest similar diagnostic accuracy for CNB due to improved histopathological procedures [8,9]. Therefore, the aim of this study was to analyse and compare the diagnostic accuracy of percutaneous CNB and open biopsy in suspected malignant primary tumours of the musculoskeletal system.

## Methods

### Patients

All patients who underwent either a percutaneous or an open biopsy, and received subsequent definite tumour resection in our musculoskeletal tumour centre between January 2007 and December 2008 were enrolled. Afterwards, this patient sample was screened for primary bone and soft tissue tumours based on the pathological report from subsequent tumour resection. Exclusion criteria were suspected benign lesions, excisional biopsy, secondary tumours and non-surgical treatment following biopsy. Indication for either procedure was based on suspected entity, radiological findings and location of the lesion.

### Biopsy techniques

The approach for biopsy was always defined by an expert orthopaedic tumour surgeon considering the subsequent resection. The percutaneous biopsy was radiologically guided either by sonography of soft-tissue masses or by CT of bone tumours, and was carried out using a 14-gauge core needle. Three to five passes were taken to obtain multiple samples throughout the tumour without breaking the far wall. Additionally, a small cutaneous incision was performed to mark the biopsy canal for the following resection. The open biopsy was performed by an expert orthopaedic tumour surgeon in line with the planned resection and according to sarcoma principles [5]. The samples were taken from the periphery of the tumour due to the frequent presence of central necrosis.

The obtained tissue was stored on ice and immediately transferred to our pathological institute for further analysis. All specimens were routinely stained with H & E. Furthermore, histochemical and special immunohistochemical stains were applied if appropriate.

### Histology report

The review of the sections was performed by two senior pathologists who specialised in the field of orthopaedic oncology. All specimens were evaluated for the nature of the lesion (benign or malignant) and the specific histological diagnosis.

### Statistics

Histopathological results from biopsy and subsequent tumour resection were compared. Sensitivity, specificity positive predictive value (PPV), negative predictive value (NPV) and diagnostic accuracy were calculated. Further analysis was performed using SPSS software (IBM, Armonk, NY, USA) and Fisher’s exact test with a 95% confidence interval.

## Results

In total, 77 patients underwent open biopsy (n = 31) or percutaneous CNB (n = 46) in our study (Table 1). A total sensitivity of 96.9% was determined for open biopsy and 88.8% for CNB (*P* = 0.28). Specificity and the PPV were 100% for both biopsy techniques. Differences in favour of open biopsy were identified for NPV (94.1% vs. 83.3%, *P* = 0.55) and diagnostic accuracy (92.9% vs. 98.0%, *P* = 0.55). The correct histopathological diagnosis compared to the subsequent resection specimen could be obtained in 93.9% after open biopsy and in 84.2% after percutaneous CNB. The difference was not statistically significant (*P* = 0.34) (Table 1).

**Table 1:** Cumulative accuracy of open biopsy compared to core needle biopsy (CNB) in bone and soft tissue tumours based on the final diagnosis

	**Percutaneous CNB (n = 46)**	**Open biopsy (n = 31)**
**Sensitivity**	88.8% (*P* = 0.28)	96.9%
**Specificity**	100%	100%
**Positive predictive value**	100%	100%
**Negative predictive value**	83.3% (*P* = 0.55)	94.1%
**Diagnostic accuracy**	92.9% (*P* = 0.55)	98.0%
**Specific diagnosis**	84.2% (*P* = 0.34)	93.9%

*P*-values are shown for comparison between CNB and open biopsy (*P* < 0.05 was considered statistically significant).

Insufficient sampling occurred in CNB of one soft tissue tumour (7.6%). During subsequent open biopsy, the correct histopathological diagnosis could be obtained. Furthermore, three biopsy specimens, one obtained by open biopsy in a bone lesion and two by CNB in soft tissue masses, were diagnosed as benign tumours but revealed malignancy after final resection. The bone lesion was initially classified as an enchondroma but turned out to be a low-grade chondrosarcoma in the resection specimen. Similarly, two lipomatous soft tissue masses were diagnosed as lipoma whereas the subsequent resection disclosed a low-grade liposarcoma in both cases. However, all tumours were resected according to sarcoma principles. Thus, all three patients with the incorrect diagnosis of a benign tumour were adequately treated with a complete resection.

We observed impaired wound healing following one open biopsy in our study. Significant complications, such as haematoma or wound infection causing morbidity that required intervention, or compromised the treatment outcome, did not occur in the CNB or open biopsy patient group.

### Bone tumours

In total 48, patients with bone tumours were included (Table 2). Open biopsy was performed in 15 and CNB in 33 patients, respectively. Sensitivity, specificity, PPV, NPV and diagnostic accuracy were 100% for CNB. Slightly inferior results were obtained with open biopsy for sensitivity (95.5%), NPV (91.7%) and diagnostic accuracy (93.3%), but the differences in values were not statistically significant (*P* > 0.05) .

**Table 2:** Accuracy of open biopsy compared to core needle biopsy (CNB) measured separately for bone and soft tissue tumours based on the final diagnosis

	**Percutaneous CNB**		**Open biopsy**	
	**Soft tissue sarcoma (n = 13)**	**Bone sarcoma (n = 33)**	**Soft tissue sarcoma (n = 16)**	**Bone sarcoma (n = 15)**
**Sensitivity**	81.8%	100%	100%	95.5%
**Specificity**	100%	100%	100%	100%
**Positive predictive value**	100%	100%	100%	100%
**Negative predictive value**	50%	100%	100%	91,7%
**Diagnostic accuracy**	84.6%	100%	100%	100%

### Soft tissue tumour

In 29 patients with soft tissue tumour, 16 open biopsies and 13 CNBs were performed (Table 2). Here, open biopsy revealed superior but not statistically significant results, for sensitivity (100% vs. 81.8%, *P* = 0.48), NPV (100% vs. 50%, *P* = 0.17) and diagnostic accuracy (100% vs. 84.6%, *P* = 0.19) compared to CNB. Specificity and PPV were 100% for both biopsy techniques.

## Discussion

In recent studies, diagnostic accuracy ranges from 74% to 98% for CNB of bone and soft tissue tumours [8-13]. In line with these results, in our study we identified an overall diagnostic accuracy of 92.9% in soft tissue tumours and 98% in bony lesions, although many authors also included metastatic tumours, usually yielding higher accuracy than more heterogeneous sarcomas [14,15]. However, Sung *et al*. found no significant differences regarding diagnostic accuracy between CNB of heterogeneous and homogeneous bone tumours [16]. The correct histopathological diagnosis compared to the resection specimen was obtained in 84.2% of soft tissue masses and 93.9% of bony lesions in our study, confirming previously published results [8,9,17].

Comparing open biopsy and CNB in suspected malignant bone tumours, we identified slightly superior results for CNB. However, considering the relatively small number of cases included in our series, the results should presumably be almost equal in a larger patient sample. CNB of soft tissue tumours revealed inferior results compared to open biopsy in the current study. These findings correlate well with recent literature, indicating that very heterogeneous tumours including liposarcoma, angiosarcoma and synovial sarcoma are, amongst others, potentially difficult to diagnose by CNB [9,16,17]. In this context, Kasraeian *et al*. found significantly deficient results regarding specific diagnosis in a prospective study comparing open biopsy with CNB in soft tissue tumours [18]. Favourable results were obtained, and there was only one non-diagnostic sample in our study (7.6%). However, this is presumably attributed to our relatively small patient sample.

To prevent lower diagnostic accuracy due to insufficient or inadequate sampling in CNB, McCarthy proposed a full discussion between orthopaedic surgeons, radiologists and pathologists to identify suitable tumour regions prior to CNB [15]. Additionally, the procedure should be conducted by a well-trained interventional radiologist or orthopaedic surgeon [4,7,19,20]. Regardless of meeting either recommendation, one CNB sample was insufficient in our series emphasizing the importance of harvesting tissue from multiple passes within the tumour.

A major disadvantage of CNB, apart from insufficient sampling, is the fixation of the tissue in formalin, as pathologic analysis is mostly limited to histology and immunohistochemistry, and frozen samples are needed for recent molecular diagnostic approaches such as real-time PCR (rt-PCR) or microarray analysis [21,22]. Furthermore, the acquisition of additional tissue for a cryobank or research purposes is limited with CNB.

On the other hand, CNB is associated with less morbidity and fewer complications compared to open biopsy. In recent studies, complication rates for open biopsy ranging from 0% to 17% are quoted [4,7,12,18,19]. Core needle biopsy, in contrast, has a reported complication rate of 0% to 7.4% [7,8,11,12,18,23,24]. Most commonly, haematoma, bleeding and infection are mentioned [12,25,26]. In our series there was no significant complication associated with either biopsy technique. However, it has to be noted that all open biopsies were performed according to the sarcoma principles with intensive haemostasis and application of a redon drain [5].

Overall, we identified good results for both biopsy techniques. However, we have to note some limitations of our study mainly due to the retrospective design. First, we were not able to compare the pathological grading of biopsy samples to the resection specimens, because grading was not performed in every case for two main reasons. One problem was possible false negative results if a low-grade area within these very heterogeneous tumours was sampled. Furthermore, grading of tumour samples harvested after neoadjuvant chemotherapy may be misleading and thus, is rarely conducted. A subsequent grading for this study was not possible in these cases. Second, we performed only one kind of biopsy technique in each patient. The indication for either procedure was made by a well-trained senior orthopaedic surgeon based on the clinical and radiological findings as well as his experience. Accordingly, there is a possible bias in favour of core needle biopsy. Third, we investigated a quite heterogeneous study population in terms of tumour entities. Although we only included patients with suspected sarcomas, great differences remain regarding histopathological analysis. Furthermore, the diagnostic standard by which both techniques were judged was based on complete surgical resection and the final clinical diagnosis of the orthopaedic oncologist. Although this is a good measure, a possibly wrong diagnosis could influence our results.

## Conclusion

In our study, moderately inferior results were found for the percutaneous biopsy technique compared to open biopsy of soft tissue tumours, whereas almost equal results were obtained in bone tumours. Thus, CNB is a safe, minimally invasive and cost-effective technique for diagnosis of bony lesions. In soft tissue masses, the indication for percutaneous CNB needs to be made carefully by an experienced orthopaedic oncologist with respect to suspected entity, extent of necrosis and location, to avoid incorrect or deficient results.

## Abbreviations

CNB: core needle biopsy; FNA: fine needle aspiration; H & E: haematoxylin and eosin; NICE: National Institute for Health and Clinical Excellence; NPV: negative predictive value; PPV: positive predictive value; rt-PCR: real-time polymerase chain reaction.

## Competing interests

All authors declare that they have no competing interests.

## Authors’ contributions

FP: conception, study design, data acquisition and interpretation, manuscript draft; CK: conception, study design, manuscript revision; UL: data acquisition; JS: data analysis and interpretation; HR: manuscript revision, final approval; RvE-R: manuscript revision, final approval. All authors read and approved the final manuscript.
